# Author's Reply: Expanding the Scope: Reflections on Digital Smoking Cessation Strategies for Diverse Age Groups

**DOI:** 10.2196/67749

**Published:** 2024-11-18

**Authors:** Margaret C Fahey

**Affiliations:** 1 Department of Psychology Middle Tennessee State University Murfreesboro, TN United States

**Keywords:** digital smoking cessation, age group comparisons, behavioral health intervention, older adult, cigarette, tobacco, quitting, telehealth, behavioral health, public health

We thank the authors for their positive response to our paper [1], which provided thoughtful comments and suggestions for future research [2]. First, the authors mention the importance of expanding the scope of our research to include younger age groups. We agree that smoking cessation is an important public health initiative across the entire lifespan. We focused on an older adult (≥65 years) population not to discount the importance of expanding digital cessation treatment among younger adults, but rather to focus on an underserved population in the field of tobacco cessation. For decades, older adults have been ignored by antitobacco public health initiatives, given a focus on tobacco prevention in adolescence and ageist misconceptions that cessation does not benefit health in later life [3]. Hence, older adults have historically been less likely to receive evidence-based cessation treatment [3]. As the field of tobacco cessation treatment is evolving to include digital platforms, older adults continue to be ignored. To our knowledge, no digital cessation treatment is tailored to the unique needs of this age group, despite numerous programs tailored specifically for adolescents or young adults [4]. Although younger age groups are generally more likely to interact with technology, older adults are using technology for their health at increasing rates [5] and should not be excluded from digital cessation treatment research. Our findings are hypothesis-generating and provide recommendations for being more inclusive of older age groups in the development of these treatments. Further, we hope that our study highlights an underrepresented voice that needs better inclusion in the field of tobacco cessation.

Second, the authors suggest that integrating social and emotional support in digital platforms, alongside traditional face-to-face counseling, could offer a comprehensive approach to cessation for this age group. We agree that incorporating social components, such as group chats within app-based programs or telehealth group counseling, might be appealing and beneficial approaches for older adults. When prompted about group-based components in digital platforms, our study population believed that the benefits of doing so would include learning new strategies for quitting and connecting with other older adults attempting to quit cigarette smoking. However, our sample discussed concerns about interpersonal challenges (eg, conflicts and negativity) among individuals, which many had witnessed on social media platforms. Perhaps, moderated or asynchronous interactions in group-based digital platforms might be more appealing for this age group. However, given ageist misconceptions that older adults are unable or unwilling to quit cigarettes [3], this population might uniquely benefit from interacting with others in their age group who are motivated to quit smoking. We thank the authors for this comment and believe that future research could consider evaluating the effectiveness of group-based digital cessation treatment for older age groups. In summary, our study challenges the bias that older adults are unwilling or uninterested in engaging with digital platforms to aid with smoking cessation. We are encouraged by the commentary that our study is eliciting, and we hope that researchers and clinicians working with older adults might benefit from our findings.
